# A geospatial investigation of microplastics leaching in Ubon Ratchathani province, Thailand: fuzzy logic-based analysis

**DOI:** 10.1007/s10661-025-14263-4

**Published:** 2025-06-30

**Authors:** Ekisha Sharma, K. C. Surendra, Dan Tran Thanh, Thammarat Koottatep

**Affiliations:** 1 Environmental Engineering and Management, School of Environment, Resources and Development, Asian Institute of Technology, Khlong Luang District, Pathum Thani, 12120 Thailand; 2 Present Address: Civil and Environmental Engineering, School of Engineering, The Hong Kong University of Science and Technology, Clear Water Bay, Kowloon, Hong Kong; 3 Department of Molecular Biosciences and Bioengineering (MBBE), University of Hawai’i at Mānoa, 1955 East-West Road, Honolulu, HI 96822 USA; 4 Global Institute for Interdisciplinary Studies, Kathmandu, Nepal; 5 GLODAL Inc., Sakuragi Cho, Naka-Ku, Yokohama, Japan

**Keywords:** Microplastics, Microplastics leakage, Fuzzy logic, Sources hotspots, Overlay analysis

## Abstract

Microplastics pollution poses significant environmental challenge, with river networks serving as major pathways for transport to oceans. Effectively managing microplastics requires identification of their sources and pathways into river networks, yet there is a lack of understanding, hindering successful mitigation efforts. This study demonstrates the novel use of fuzzy logic-based tools in geographic information system (GIS) for the precise identification of microplastics leakage sources in Ubon Ratchathani province, situated in Northeastern Thailand. A leakage density map was developed by applying fuzzy logic to variables responsible for microplastics production in the environment, using available geospatial datasets. A fuzzy overlay was performed, merging the density map and drainage networks of the province, creating a comprehensive microplastics leakage sources map. This leakage sources map illustrated the flow of microplastics from leakage-dense areas towards the susceptible river network in the province. It identified key sources of microplastic leakage, such as road networks, facilities, and industries contaminating urban waterways. Field-based microplastic data verified the map’s accuracy. A comparative analysis between identified polluted rivers and those not flagged revealed that microplastic accumulation is influenced not only by source proximity but also by river characteristics such as flow rate, hydrology, and seasonal variations. The study underscores the effectiveness and reliability of fuzzy logic-based GIS tools in identifying microplastics source hotspots within a specific region. Furthermore, it provides a valuable approach for advancing Sustainable Development Goal 14 (Life Below Water) by managing microplastics in river networks to prevent their accumulation in marine environments.

## Introduction

Plastics have become an indispensable part of modern society, leading to an unprecedented surge in plastic production and use; however, their mismanagement has resulted in far-reaching environmental consequences. In 2019, global plastic production reached 353 million metric tons, with only 9% being recycled and 19% combusted for energy recovery (OECD, 2021). However, a significant portion of plastic waste, 50%, is still being landfilled. Additionally, 22% of plastic waste is not processed through the current waste management system, ending up in unregulated dumpsites, where it may be incinerated or discharged into nearby water bodies (OECD, 2021). These practices have led to the accumulation of plastics and microplastics in various ecosystems, highlighting the inability of waste management practices in coping with the escalating production and utilization of plastics.

Studies have highlighted the adverse impacts of microplastics in different ecosystems, including agricultural lands (Campanale et al., 2022; Tympa et al., 2021), the human body (Zhang et al., 2022), and marine environments (Jiang et al., 2022; La Daana et al., 2022) among others. In oceans, microplastics pose a serious concern as they hinder photosynthesis in marine ecosystems, threatening global oxygen production (Sjollema et al., 2016). Microplastics also negatively impact zooplankton in the oceans, disrupting their crucial role in the flow of the food, mass, and energy in the marine ecosystems. Additionally, studies have reported presence of microplastics in the ocean beds, which can adversely impact carbon storage in deep waters (Shen et al., 2020).

The presence of plastics and microplastics in marine ecosystems is not solely attributed to the anthropogenic activities in coastal regions but are also an outcome of extensive river networks acting as a conduit for waste from deep inlands to the oceans (Harris et al., 2021). In fact, more than a thousand rivers worldwide are responsible for 80% of plastic discharges into the oceans (Meijer et al., 2021). Globally, 0.8–2.7 million metric tons of plastic waste is released from river networks per year (UNEP, 2022), among which, the lower Mekong, draining from Northeast Thailand, has been identified among the top ten major contributors (MRC, 2022; Tran-Thanh et al., 2022; UNEP, 2023). Studies have also identified microplastics in Thailand’s major river networks, such as Chao Phraya (Oo et al., 2021; Ta & Babel, 2020) and U-Taphoe (Pradit et al., 2023).

Within river networks, microplastics serve as a novel breeding ground for native microbial communities, inducing changes in their structure and composition (Citterich et al., 2023). Since native microbial communities form the foundation of local ecosystems, any shifts in their structural composition threatens the existing ecological balances. Additionally, microplastics act as a carrier for toxins in freshwater bodies due to their ability to adsorb heavy metals and persistent organic pollutants, such as polychlorinated biphenyls and polychromatic aromatic hydrocarbons (Talvitie et al., 2017; Rose et al., 2023).

To alleviate the risks of microplastics to river and marine ecosystems, an in-depth understanding of their distribution in such ecosystems is crucial. Plastics or microplastics leakage hotspots are components of any system that directly and/or indirectly contribute to such leakage, offering important information about the potential sources, fate, and transport dynamics of plastics or microplastics in different ecosystems (Boucher et al., 2020; Nel et al., 2020). These hotspots help in identification of vulnerable ecosystems and provide a clear understanding of the specific risks posed to them (Nel et al., 2020; Underwood et al., 2017).

Leakage hotspots can be geographic locations (i.e., regional hotspots), elements of the plastic value chain, waste management hotspots, industrial sector hotspots, or plastic application hotspots. Regional hotspots are areas that have the highest potential of leaking plastics or microplastics into the waterways within a country or region. Generally, regional hotspots can be identified using geographic information system (GIS), a powerful set of tools which can analyze spatial data in layers, where the hotspots are shown on a map (Baigorria & Romero, 2007; Boucher et al., 2020). These maps draw a clear image of plastics or microplastics leakage in specific regions or countries, thus serving as an effective tool for regulatory bodies to implement policies and/or practices to mitigate plastics pollution.

Rinasti et al. (2022) successfully identified plastics leakage hotspots in Jakarta and Bandung, Indonesia, by using an integration of geospatial analysis and multi-criteria decision making (MCDM)-based material flow analysis. The combination of GIS-MCDM tools are a valuable approach for assessing different types of environmental problems and help make informed decisions (Jelokhani-Niaraki et al., 2018). Studies have identified plastics leakage hotspots in various locations in Southeast Asia (Chukwuma et al., 2019; Tran-Thanh et al., 2022) and Africa (Chukwuma et al., 2021) using GIS-based fuzzy overlay analysis. Fuzzy overlay analysis, based on fuzzy logic, incorporates the inherent uncertainty and subjective perceptions of factors affecting any decision in a real-world situation (Mahjouri et al., 2017; Nadiri et al., 2018).

For the purpose of plastics pollution research, fuzzy overlay analysis incorporates the indefiniteness of the various factors that cause such pollution. For example, Tran-Thanh et al. (2022) incorporated slope as one of the fourteen variables to identify plastics leakage hotspots using fuzzy overlay analysis. Human settlement and activities, which are responsible for plastic waste generation, tend to be concentrated in areas with low terrain. As a result, plastics leakage is more likely to be higher in these lower terrains. However, it is difficult to determine the crisp value of the slope of the terrain at which plastics leakage exactly declines. It can be said that the smaller the slope of the terrain, the higher the probability of plastics leakage. Fuzzy logic allows us to use this relationship of slope with plastics leakage as an input to identify leakage hotspots.

Despite being used to identify plastics leakage hotspots, fuzzy logic-based GIS tools have not, to the best of authors’ knowledge, been used for identifying microplastics leakage hotspots. The increasing concern of microplastic pollution highlights the necessity of pinpointing the areas where they are most likely to enter rivers and developing effective measures to reduce their flow into waterways. Previously, fuzzy logic-based GIS tools have been proven effective in identifying plastics leakage hotspots by accounting uncertainties of variables that contribute to plastics production (Chukwuma et al., 2019, 2021; Tran-Thanh et al., 2022). Such advantages of fuzzy logic can also be utilized in determining microplastics leakage hotspots by incorporating subjective variables that contribute to microplastics pollution.

A novel application of fuzzy logic-based GIS tools was used to identify microplastics leakage hotspots in Ubon Ratchathani, a province located in Northeastern Thailand, covering a significant area of the lower Mekong region, and identified as a large contributor to plastics entering the oceans (Tran-Thanh et al., 2022). Fuzzy logic-based GIS tools were used to generate maps that highlighted the sub-districts of Ubon Ratchathani with the highest potential for microplastics leakage as well the local river networks that are highly susceptible to such pollution. The identified hotspots were validated by a field-based study conducted during different seasons from 2019 to 2021. Additionally, the data of microplastics found in the river inside the hotspot (Mun River) was compared with the one that was outside the hotspot (Phap River). The obtained microplastics leakage map can serve as a starting point for designing efficient strategies and mitigation plans for microplastics pollution control in the province and beyond. This initiative aligns with Sustainable Development Goal 14, (Life Below Water) by focusing on reducing riverine plastic pollution that eventually impacts marine ecosystems, thus helping to conserve and sustainably use aquatic resources.

## Methods

**Fig. 1:**
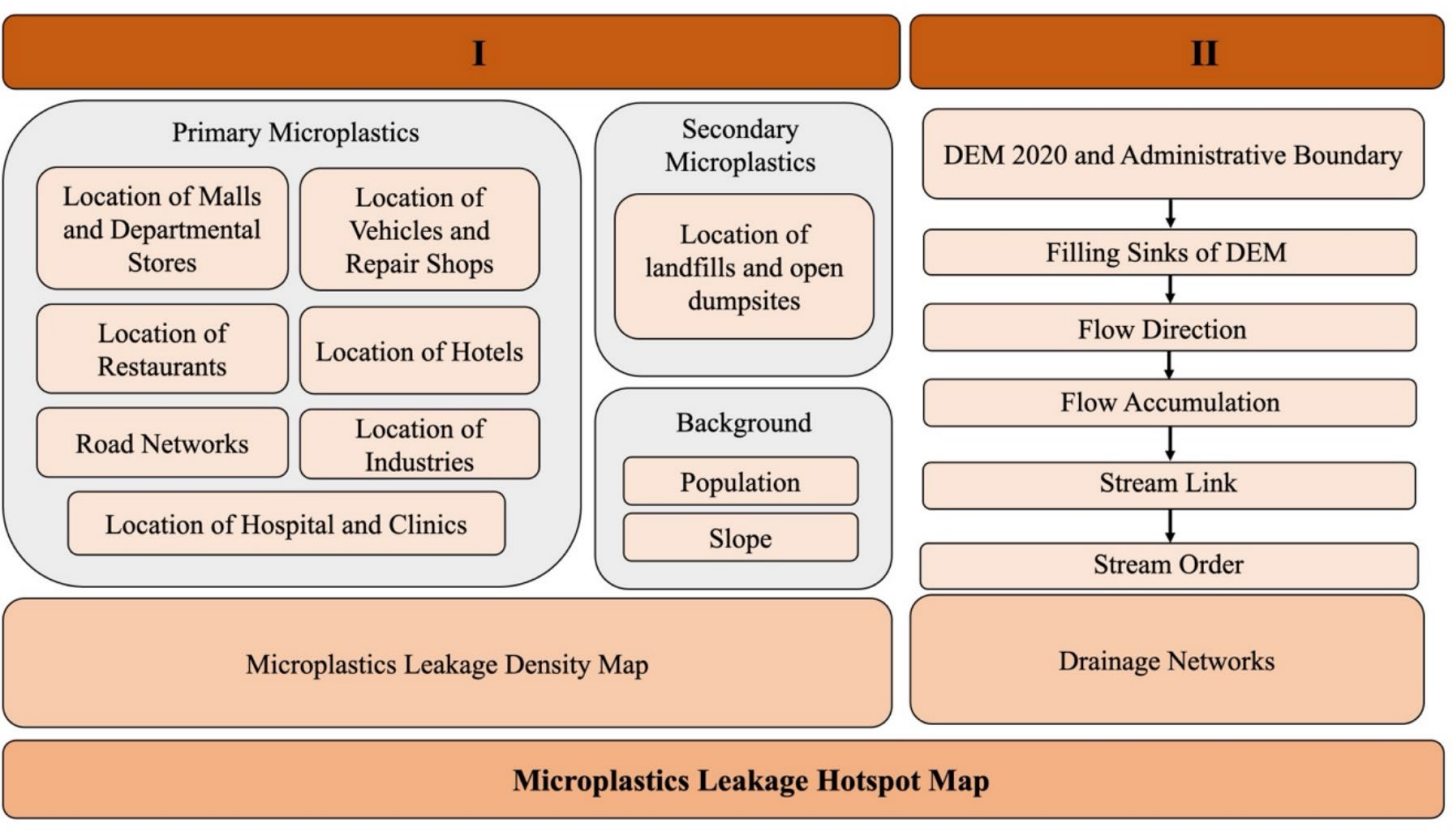
Two-step methodology for developing microplastics leakage hotspot map followed in GIS

Identification of regional hotspots can provide a comprehensive view of the sources of microplastics leakage into the waterways of the selected area. This study introduces a novel application of fuzzy logic-based GIS tools, a method not previously explored in microplastics analysis, to precisely map and identify areas with the highest potential for leakage, offering new insights into environmental management and pollution mitigation. Two major steps were followed to obtain the regional hotspots for Ubon Ratchathani, Thailand: (i) creating a microplastics leakage density map and (ii) overlaying the obtained density map on the drainage networks to generate a hotspot map, which is shown in Fig. 1. In the first step, variables responsible for production of primary and secondary microplastics were enlisted. In GIS, these variables are locations of sources responsible for microplastics generation. Fuzzy overlay analysis was performed by first fuzzifying these locations, followed by assigning a membership function based on their effect on the outflow of microplastics, and then finally defuzzifying them to be showcased on a map. In the second step, a Digital Elevation Model (DEM), 2020, was used to obtain drainage networks of Ubon Ratchathani. These drainage networks were then overlain, using fuzzy overlay parameters, onto the previously obtained microplastics leakage density map to produce the final microplastics leakage hotspots map. Further details regarding the methodology are discussed in the following sections.

### Study area

**Fig. 2:**
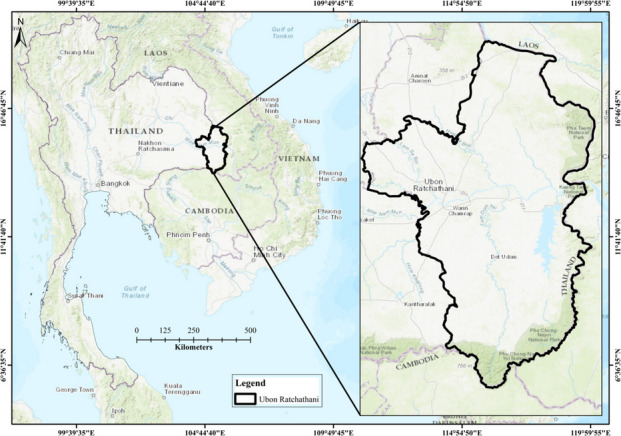
Study area: Ubon Ratchathani province, Thailand

The study was conducted on Ubon Ratchathani province, located in the Isan region of Northeastern Thailand, as shown in Fig. 2. Over 1600 metric tons/day of municipal solid waste (MSW) was generated in Ubon Ratchathani province in 2020 (TPCD, 2020; Tran-Thanh et al., 2022). About half of the total MSW was disposed of either in two sanitary landfills, located in the Warin Chamrap and Phibun Mangsahan districts, or into one of 28 unregulated dumping sites distributed throughout the province.

### Microplastics leakage density map generation

**Fig. 3:**
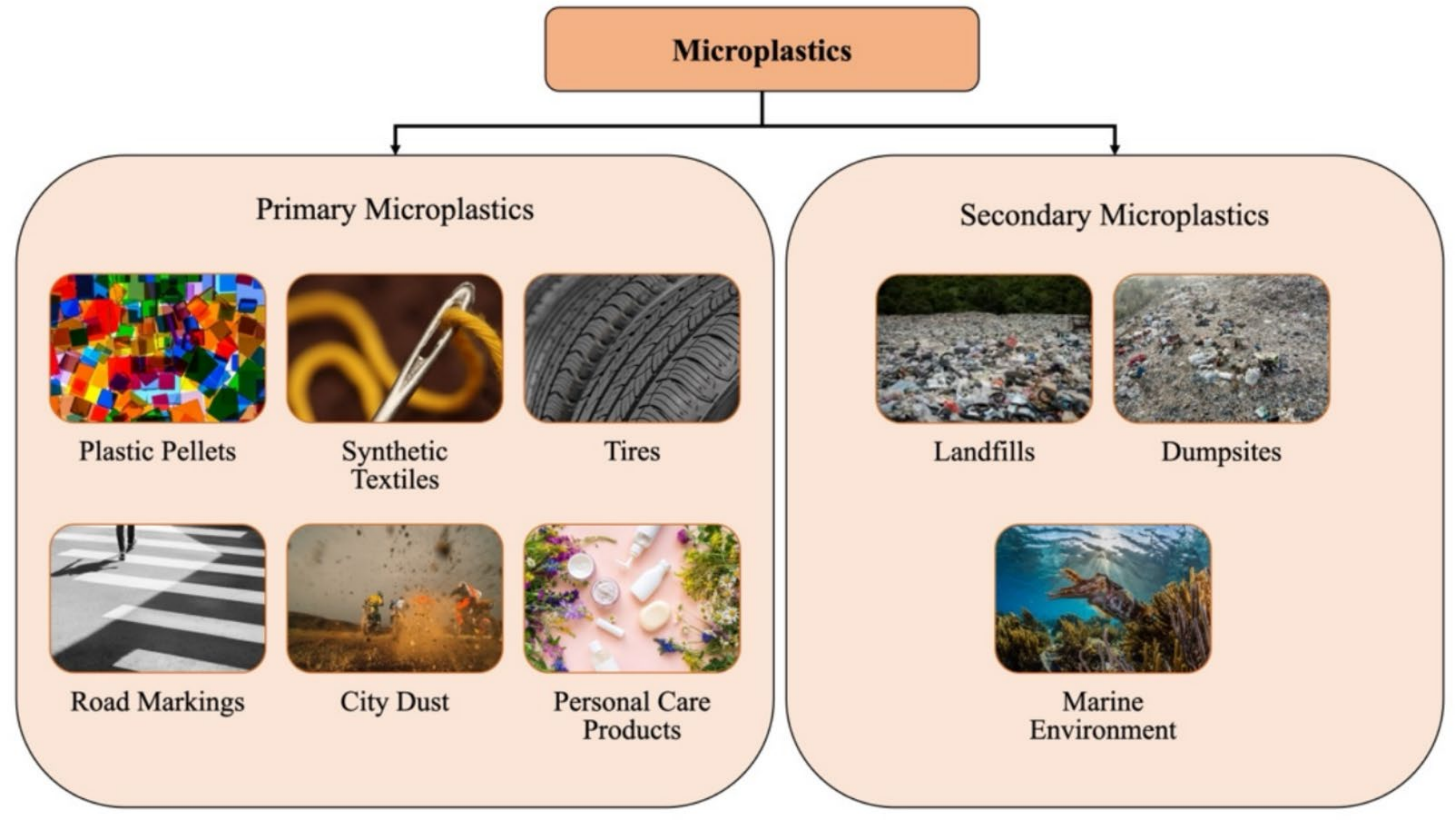
Primary and secondary sources of microplastics

To create a microplastics leakage density map, all variables that are highly correlated with production and distribution of microplastics were listed. These variables were selected as indicators of microplastic production and highlight instances where microplastics could potentially leak into the environment. Variables were selected following a similar approach as reported in previous studies on plastics leakage (Boucher & Friot, 2017; Tran-Thanh et al., 2022). Based on the mode of microplastics production, variables were categorized into two groups: “primary” and “secondary,” which is shown in Fig. 3.

Primary microplastics are the ones that are specifically manufactured for either industrial or household purposes (Krishnan et al., 2023). There are six types of primary microplastics: plastic pellets, synthetic textiles, tires, road markings, city dust, and personal care products (Boucher & Friot, 2017; van Wezel et al., 2016; Wang et al., 2019). City dust comprises nine sources of microplastics in the urban environment: abrasion of synthetic soles of footwear, synthetic cooking utensils, artificial turfs, building coatings, household dust, city dust, infrastructure such as harbors and marina, blasting of abrasives, and detergents (Essel et al., 2015; Lassen et al., 2015; Sundt et al., 2014).

Secondary microplastics are degraded plastic wastes in landfills, dumpsites, and marine environments (Jaikumar et al., 2019; Krishnan et al., 2023). Through runoff, wastewater, and wind, these microplastics reach water networks, and eventually the oceans (Boucher & Friot, 2017; Oladoja & Unuabonah, 2021). In this study, an additional group of variables, “background,” comprising of variables, such as population density and terrain slope was also used. All selected variables along with their respective data sources used for the fuzzy overlay analysis are summarized in Table 1.

**Table 1:** Selected variables for fuzzy overlay analysis

Group of variables	Individual variables	Datasets	
		Source	Form
Primary microplastics	Location of hotels	(Open Street Map, 2020)	Point
	Location of restaurants		
	Location of hospitals and clinics		
	Location of malls and departmental stores		
	Location of vehicles and repair shops		
	Location of industries (textiles, tires, chemicals, cosmetics, electronics, construction, and transportation)		
	Road networks (primary, secondary, and tertiary highways; service, residential, and unclassified roads)	(Open Street Map, 2020)	Line
Secondary microplastics	Location of landfills and dumpsites	(TPCD, 2020)	Point
Background	Population density	(WorldPop, 2020)	Raster
	Slope	(USGS, 2020)	

Primary microplastics: plastic pellets, synthetic textiles, tires, road markings city dust, and personal care products; secondary microplastics: plastics decomposed in landfills, dumpsites, or marine environments

The datasets utilized for GIS analysis were sourced from both open-access platforms and local governmental bodies. This dual-source approach ensured a comprehensive representation of the variables influencing microplastics leakage within the selected study area. Open-source datasets provided a broad range of ancillary data, including baseline geographical and demographic information that is essential for spatial analysis. These publicly accessible datasets offer the advantage of flexibility and ease of integration into various analytical frameworks, while also enabling transparency and reproducibility of the research findings. In addition, datasets obtained from local bodies contributed critical, context-specific insights that might not be available through global open-source platforms. Such data often includes fine-grained details about local environmental conditions, which are vital for a nuanced understanding of microplastics leakage dynamics. This comprehensive data foundation enhances the credibility and applicability of the results, allowing for more effective identification and management of leakage hotspots in the area.

#### Fuzzy logic and fuzzy overlay analysis in GIS

The uncertainties present in real-world data are generally expressed using terminologies, such as certainly, mostly, mainly, and rarely, among others (Angadi et al., 2021). Such terminologies lack a crisp value and can become a bottleneck if a decision needs to be made based on them. Fuzzy logic allows incorporation of such uncertainties to the decision-making process by changing ambiguous data into probability distributions in terms of memberships (e.g., linear, Gaussian, triangular, trapezoidal, and sigmoid) that can be used in a logical reasoning (Angadi et al., 2021; Zadeh et al., 1996).

**Fig. 4:**
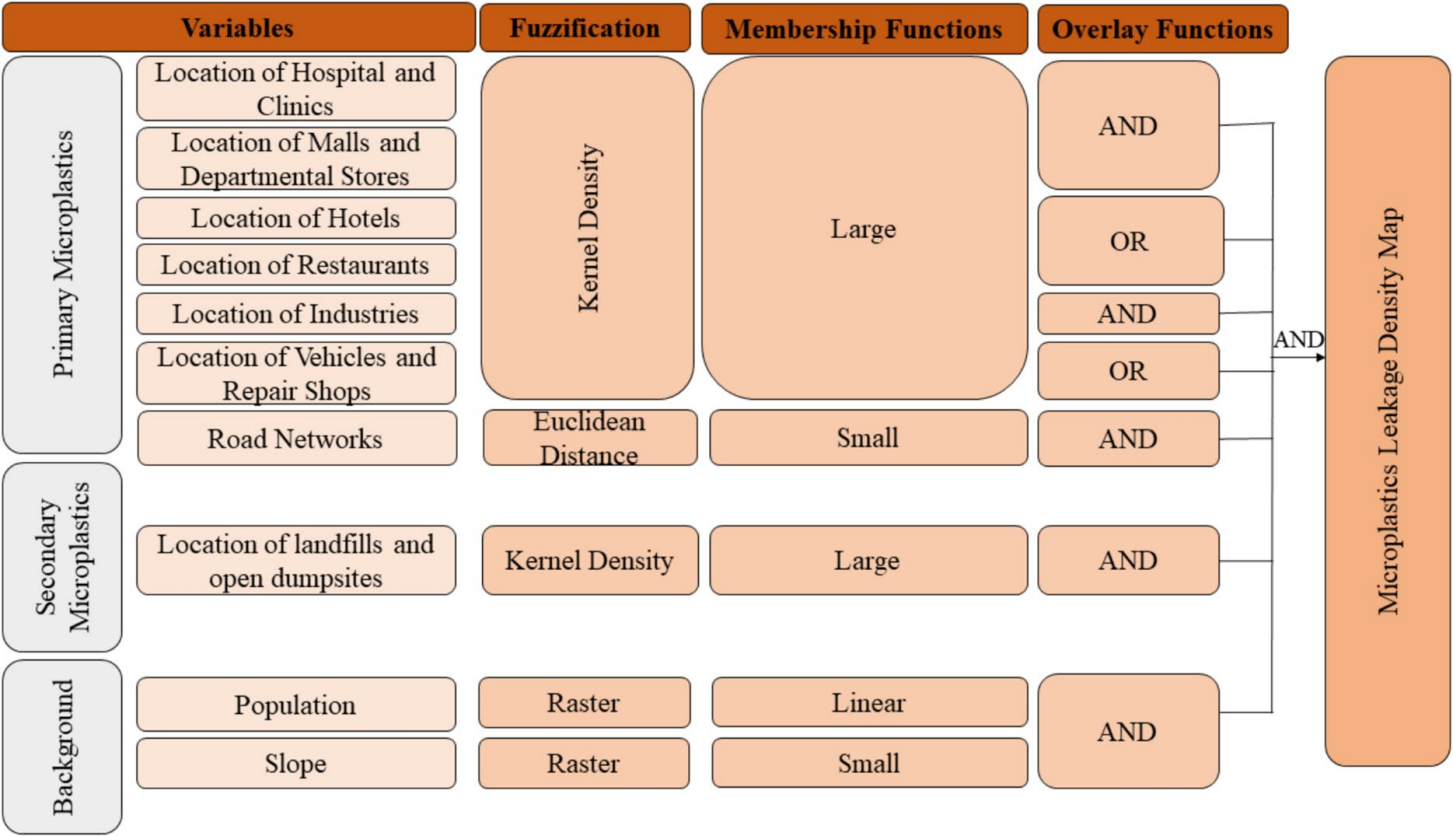
Schematics of the overall fuzzy overlay analysis performed to create a microplastics leakage density map

In GIS, fuzzy logic is used for fuzzy overlay analysis, which changes geographic data into membership functions for the decision-making process. In this study, the GIS-based analysis was conducted in three major steps: (i) fuzzification of input variables, (ii) assignment of membership functions, and (iii) fuzzy overlay and defuzzification. All variables in each group (i.e., primary, secondary, and background) were first fuzzified and then transformed into membership functions based on their roles on microplastics leakage. The details of the fuzzification and logic of assignment for each membership function are summarized in Table 2. Subsequently, these variables, along with their assigned memberships, were overlaid and defuzzified to generate a microplastics leakage density map. A schematic representation of the fuzzy overlay analysis is shown in Fig. 4.

**Table 2:** Functions used for raster conversion and membership function for variables

Variables	Raster form	Membership function	Reason
Restaurants	Kernel density	Large	The larger the number of restaurants in an area, the higher the leakage of microplastics
Hotels	Kernel density	Large	The larger the number of hotels, the higher the leakage of microplastics
Hospitals and clinics	Kernel density	Large	The larger the area that receives the flood, the larger the leakage of microplastics
Malls and departmental stores	Kernel density	Large	The larger the number of malls and departmental stores, the higher its leakage
Car, motorcycle, tire, and repair shops	Kernel density	Large	The larger the number of these shops, the higher the leakage of microplastics
Primary, secondary, tertiary highway; residential, unclassified, and service roads	Euclidean distance	Small	The smaller the distance from the road networks, the higher the leakage of microplastics
Construction, transportation, electronics, and tire industries	Kernel density	Large	The larger the number of industries producing microplastics, the higher the microplastic leakage
Landfills and open dumpsites	Kernel density	Large	The larger the number of landfills and open dumpsites, the higher the secondary microplastics leakage
Population density	Raster	Linear	As the population increases, the microplastics leakage from the area also increases due to increase in use of plastics
Slope	Raster	Small	A smaller slope favors human inhabitation which increases microplastics leakage

##### Fuzzification of input variables

Fuzzification transforms crisp values of the input variables into fuzzy values, which can then be assigned specific membership functions (Uzun et al., 2021). All the input variables (i.e., locations responsible for microplastics generation) can only be fuzzified after their rasterization. The rasterization of data converts the given variables into pixels, where each cell contains information (ESRI, 2022c). The variables that were in point and line form (Table 1) were rasterized using kernel density and Euclidean distance, respectively.

Kernel density provides the geographical density of the variables contributing to microplastics pollution. In GIS, the kernel density computes the density of the point features in the neighborhood around them. In theory, this density is calculated by using a smooth curved surface which is fitted over each of the points, where the highest value is given to the point which decreases when it goes further up to zero, building up a circular neighborhood (Silverman, 1986). The kernel density was calculated in GIS by selecting Kernel Density in Density category of the Spatial Analyst toolsets.

For the variables in line form, the Euclidean distance was calculated, which is the distance from the center of the source of the cell to the mid-point of each neighboring cell (ESRI, 2020). The shortest distance was selected to be put in the output raster (ESRI, 2020). This distance of the “line” variables was needed to analyze their likelihood of causing microplastics pollution in the river bodies. The Euclidean distance was calculated in GIS using Euclidean Distance in Distance toolset under Spatial Analyst toolbox.

##### Assignment of membership functions

GIS has eleven membership functions for fuzzy logic: “linear,” “large,” “small,” “MS large,” “MS small,” “near,” “Gaussian,” “table of contents,” “categorical,” “somewhat,” and “vary” (ESRI, 2022b). Each of the input variables were assigned memberships based on their likelihood of causing microplastics leakage. The selected membership functions illustrate the direct relationship between the variables and their correlation with microplastics, as well as the probability of indicating the extent of microplastic leakage depending on the levels of these variables. In this study, three memberships functions, namely “linear,” “large,” and “small,” were used.

The “large” function was used for most of the variables in the primary and secondary groups of microplastics, indicating that the higher the number of locations, the higher the outflow of microplastics originating from these locations. The road networks were only one variable under the primary microplastics group that was assigned the “small” function, following the logic that the shorter the distance from roads to the drainage networks, the higher the microplastics leakage. This proximity facilitates transfer of microplastics, which increases environmental contamination. For the slope variable, the “small” function was used, as areas with low elevation favor more human inhabitation, thereby increasing the number of microplastics sources. Primary microplastics are likely to be found in areas with a lower slope. For the population density, a variable under the background group, a “linear” function was applied, reflecting positive correlation between outflow of microplastics and population density. This relationship suggests that as population density increases, the potential for microplastic pollution also rises due to the greater human activity and waste generation.

In selecting the appropriate membership functions for each variable, the spread of the variables was carefully considered. The spread parameter is critical as it modulates the rate at which fuzzy membership values decrease from complete membership (1) to no membership (0) over the range of data points (ESRI, 2022b). Specifically, a larger spread results in a rapid transition, leading to a narrow band of values being considered as high membership. Conversely, a smaller spread facilitates a more gradual transition, thereby expanding the range of values that achieve significant membership.

In context of this study, the choice of a smaller spread emphasizes each location’s contribution to microplastics sources, ensuring that nearly all relevant values are considered. Opting for a larger spread might only highlight particularly high values, potentially overlooking moderate contributors. Therefore, a small spread was uniformly applied across all membership functions, as supported by ESRI (2022b), which suggests using a minimal value of 1 for both small and large membership functions. This strategic decision ensures that a broader spectrum of data values is recognized as significantly contributing to the microplastics leakage. Consequently, this approach allows for the comprehensive inclusion of each variable’s impact in the final hotspot map, enhancing the robustness and validity of the final leakage microplastics hotspot map.

#### Fuzzy overlay and defuzzification

The fuzzy overlay functions were executed after the application of the membership functions to each variable. Among the four available overlay functions in GIS (e.g., AND, OR PRODUCT, SUM, and GAMMA), the AND function was predominantly used, as it gives the least common denominator for membership (ESRI, 2022a). This allows us to assess the impact of each variable on microplastics leakage from the region. When the overlay function AND was used for variables restaurants and hotels, as well as vehicles and repair shops, a low common denominator of these variables was given indicating an overall low impact on microplastics leakage. This could be due to the difference in spatial distribution and frequency among these variables.

Alternatively, OR function gets the highest membership values among these variables allowing the inclusion of the variable that caused high microplastics leakage. This made OR function more suitable for the group of variables restaurants and hotels, as well as vehicles and repair shops, as it allowed to use the variable that caused highest microplastics leakage, rather than the AND function, which showed a low common denominator, and did not allow any of the variables to be included in the analysis, even though any one of the variables could still contribute significantly to microplastics leakage. Functions PRODUCT, SUM, and GAMMA were not applicable in this case as none showed the combined effect of all the variables.

Multiple overlays were conducted for each group of variables and then for combinations of these groups a final AND overlay was applied to obtain a final output map, showing the combined effect of all the variables in microplastics leakage. The final overlay map showed output values between 0 and 1, where 1 represents full membership and 0 represents non-membership. These values were then defuzzified to represent in the microplastics leakage density map. The values were reclassified in the GIS into five classes, namely low, very low, medium, high, and very high, representing the microplastics leakage potential. In this case, the higher membership values above 0.50 were shown as very high, while the lowest membership value obtained was 0.08, which was indicated as very low.

### Generation of microplastics leakage hotspots

**Fig. 5:**
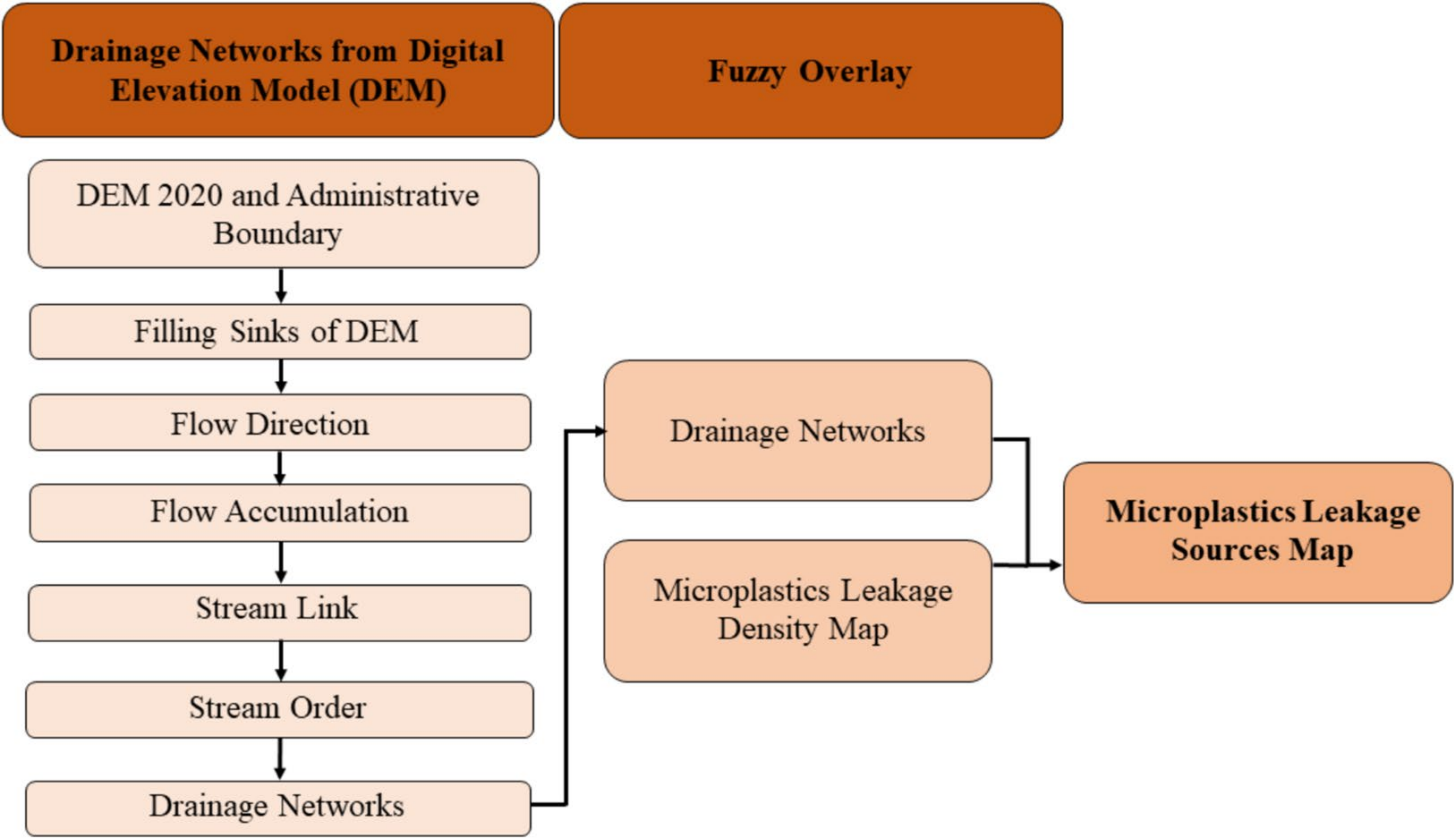
Schematics of the major steps followed for generating microplastics leakage hotspots using the drainage networks

A natural drainage network map of Ubon Ratchathani province was produced based on DEM, 2020 obtained from (USGS, 2020), using Hydrology tools under Spatial Analyst toolsets developed by ESRI. A schematic representation of major steps followed in creating microplastics leakage hotspots is shown in Fig. 5. The stream delineation was performed by filling the sinks of the DEM file, followed by finding the flow direction and accumulation. The flow accumulation was used to create a stream link, and finally delineated into stream order. The stream order was converted into features to obtain the drainage networks for the province. The resulting drainage networks in its raster form, along with the administrative network of Ubon Ratchathani province, were overlaid with the microplastics leakage density maps to produce the microplastics leakage hotspots map.

### Verification of microplastics leakage hotspots map using field study

The obtained microplastics leakage hotspots map shows the areas and rivers that may have high microplastics pollution. The presence of microplastics in the identified rivers from a field-based study will verify the reliability of these hotspots map. In this study, the map was verified using the field-based microplastics data of two rivers, one flowing inside the hotspot area (Mun River) and the other flowing outside the hotspot area (Phap River).

The data of microplastics present in Mun River was obtained from PIRIKA Inc. through personal communication, who conducted a microplastics survey in October 2019 using a device developed by themselves named Albatross Mark 5. This device consisted of plankton nets, a submersible waterproof pump, a propeller, a flow counter, and some weights to maintain stability in water (Abeynayaka et al., 2020). Albatross provides significant advantages over other sampling methods as it can filter a high volume of water in less time, i.e., 7.5–21 m^3^ of water in three minutes (Abeynayaka et al., 2020).

Microplastics in Phap River was surveyed in December 2020 and February 2021 also using another version of Albatross Mark 5 developed by Pirika Inc., Japan. This device also consists of removable plankton nets and is powered by a battery. Microplastics samples were collected from three sampling sites by submerging the device to a depth of a few centimeters for a period of five minutes. The plankton nets were then removed and immediately stored at 5 °C until further analysis. The flow meter before and after running the device was noted to calculate the volume of water filtered.

The plankton nets were washed with deionized water to extract microplastic samples. The organic contaminants present in the samples were removed by digesting the samples using hydrogen peroxide (H_2_O_2_). In brief, the sample was boiled with 20 mL of 0.05 M iron(II) and 30% H_2_O_2_ at 90 °C until all the bubbles completely disappeared, followed by heating at 75 °C for 30 min. Following the digestion process, the solution was mixed with NaCl at 6:20 ratio (i.e., 6 g of NaCl per 20 mL of sample) and heated to 75 °C until all the NaCl dissolved. Then, the solution was transferred to a glass funnel and stored at room temperature for 24 h, allowing microplastics to float. Finally, the solution was sieved through 5 mm and 0.3 mm mesh, washed off with deionized water into a petri dish.

The petri dish along with collected microplastics samples were dried at 105 °C for 24 h. The microplastics in the samples were quantified using stereomicroscope (40 × magnification). The types of microplastics were identified using attenuated total reflection–Fourier transform infrared (ATR-FTIR). Additionally, microplastics found in the Phap River was compared with the microplastics found in the rivers present inside the leakage dense areas identified in this study.

## Results and discussion

### Microplastics leakage density maps and source hotspots

**Fig. 6:**
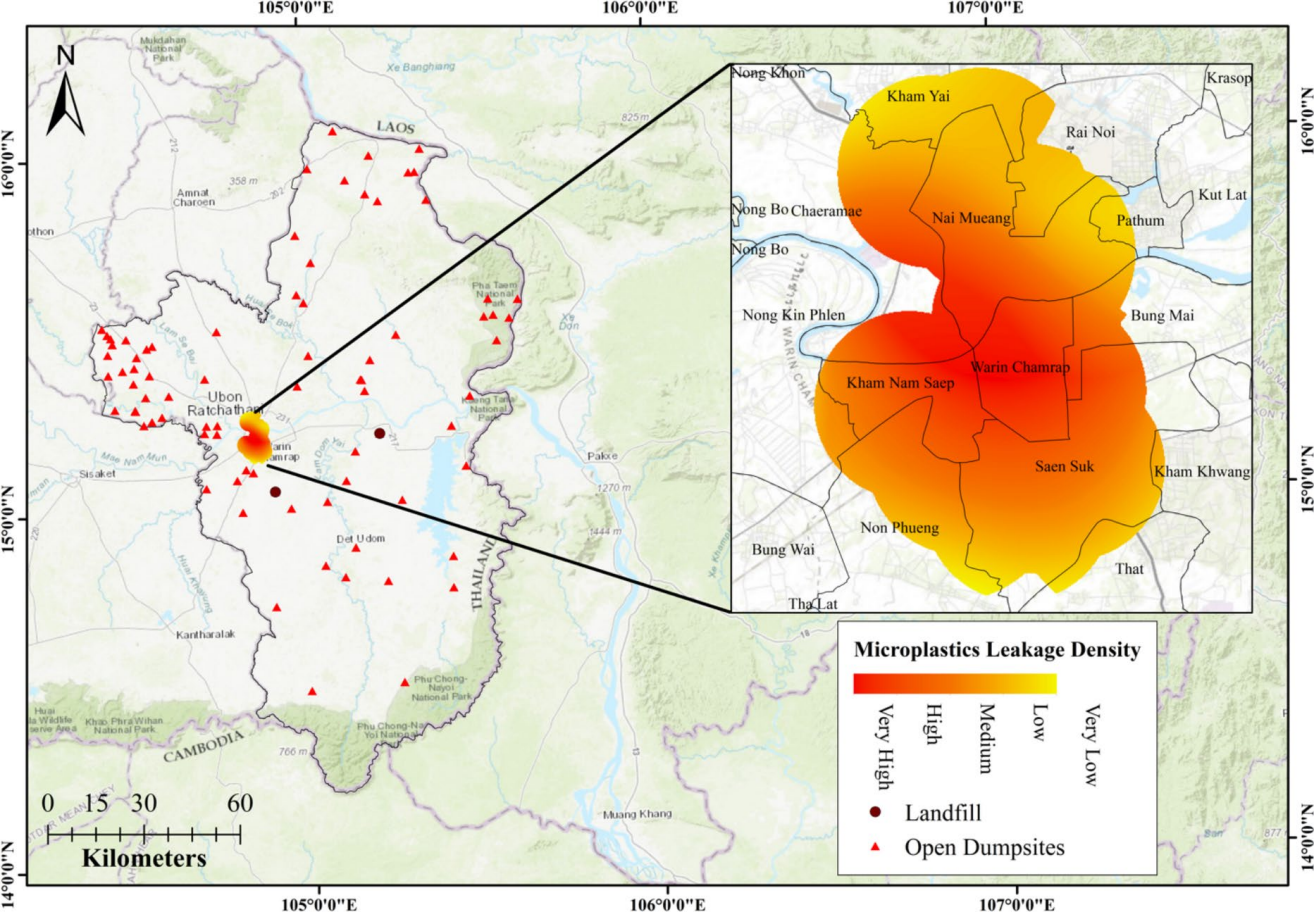
Microplastics leakage density map of Ubon Ratchathani, Thailand

**Fig. 7:**
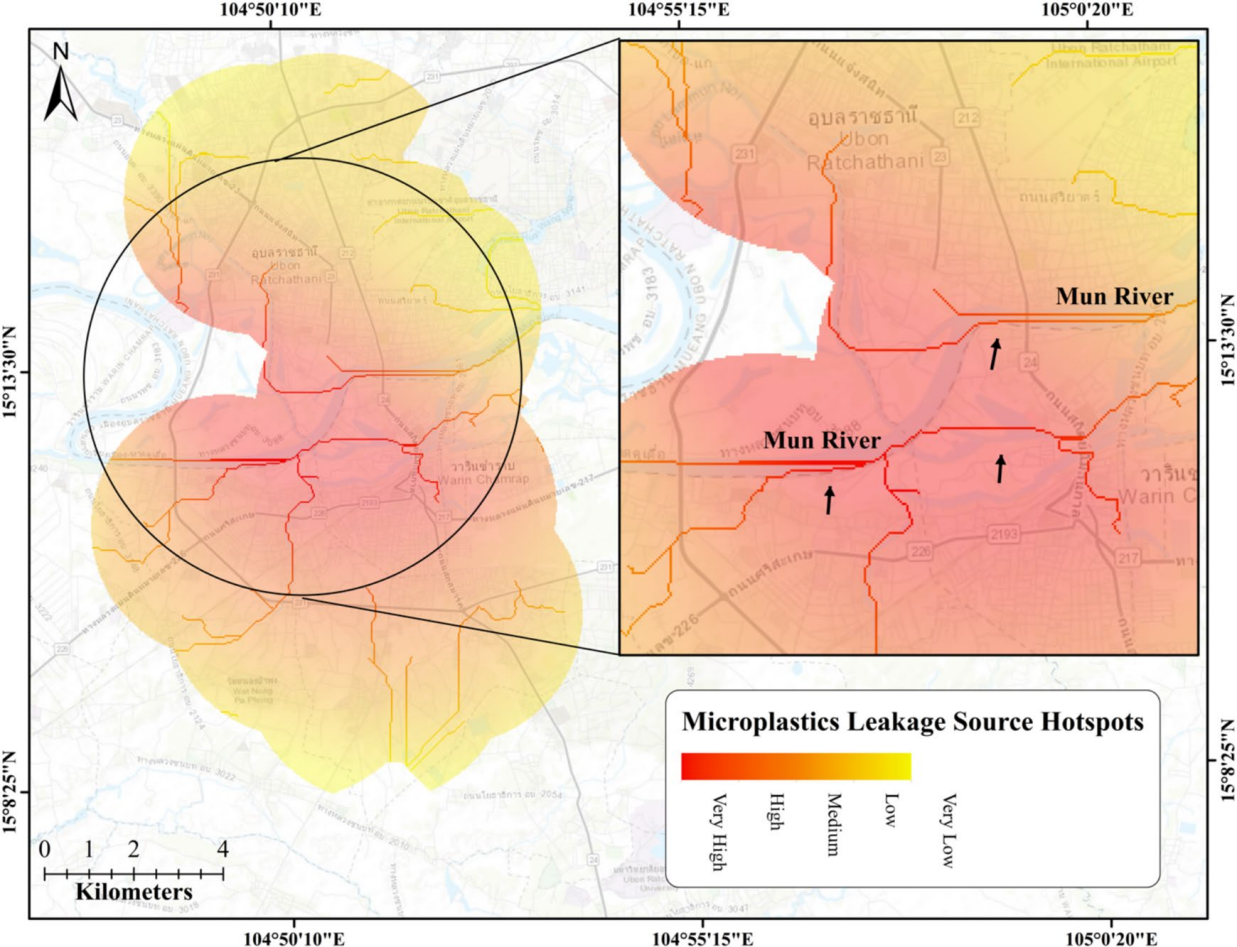
Microplastics leakage source hotspots in Ubon Ratchathani, Thailand (arrows indicates the runoff flow direction)

**Fig. 8:**
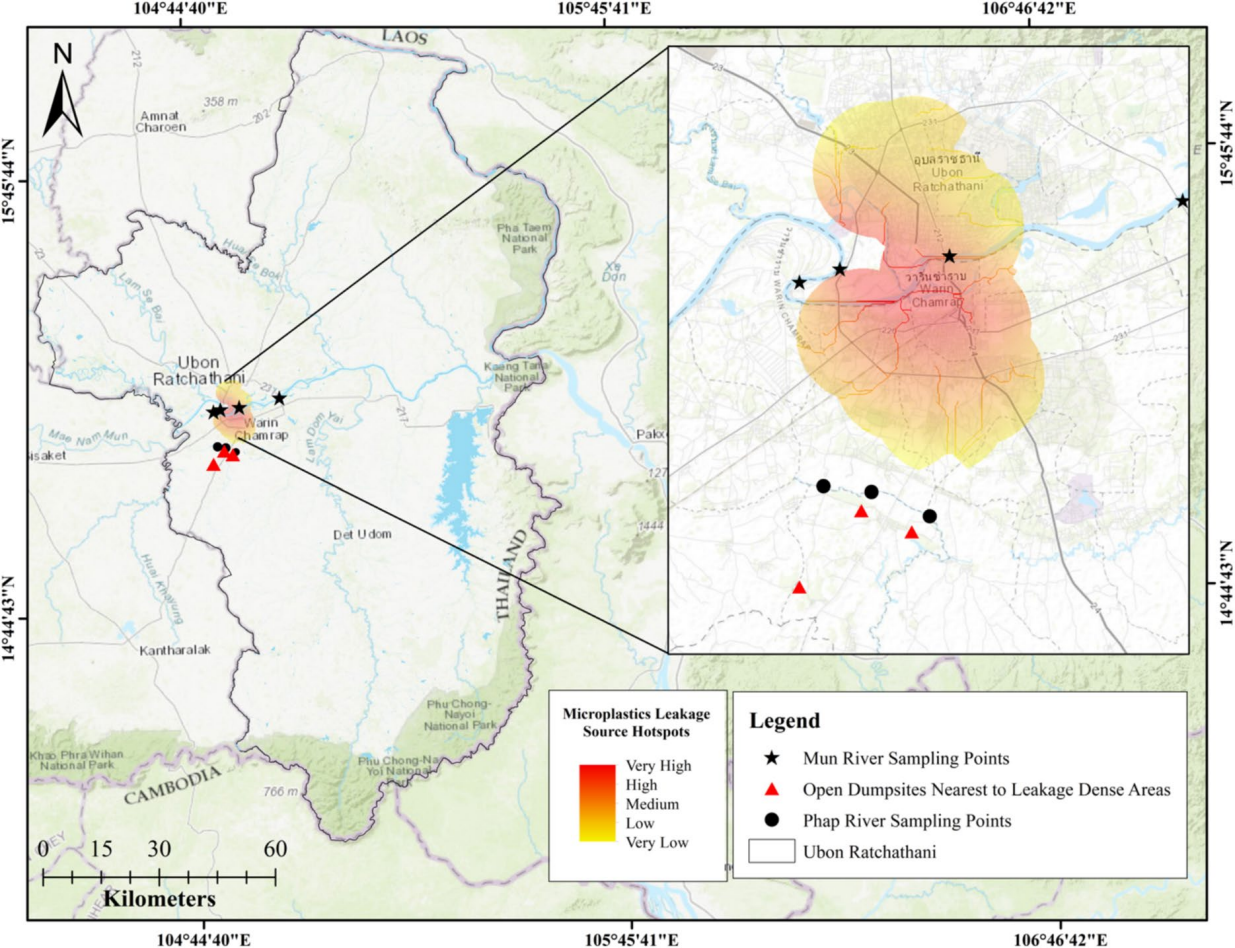
Microplastics sampling points of river bodies in Ubon Ratchathani, Thailand

This study aimed at creating microplastics leakage hotspots for Ubon Ratchathani, a northeastern province of Thailand, by using fuzzy logic-based GIS tools. The microplastics leakage hotspots were created by generating a microplastics leakage density map (Fig. 6) and a leakage sources map (Fig. 7). On both the maps (i.e., Figs. 7 and 8), different colors are used to represent five categories of leakage potential: dark red for very high, red for high, orange for medium, dark yellow for low, and yellow for very low. The microplastics leakage density map was generated based on fuzzified variables responsible for generation and distribution of microplastics in the environment. The hotspot map was obtained by overlaying the microplastics leakage density map with drainage networks obtained from the DEM, 2020. Overall, the leakage density map shows different sources contributing microplastics to the environment, while the hotspots map shows the microplastics’ flow direction into the river bodies in the same environment.

As shown in the microplastics leakage density map (Fig. 7), microplastics leakage is more concentrated to urban sub-districts of the province, contributed mainly by road networks and facilities, such as restaurants, hotels, and department stores/malls. These areas also have a high number of construction and transportation-based firms, leading to microplastics leakage into the surrounding environment. These urban sub-districts of Ubon Ratchathani are situated at low altitudes, which are generally favored for settlement due to land accessibility. This inevitably leads to wider road networks and more infrastructure and facilities to support anthropogenic activities. In general, industries are also located in urban centers due to better infrastructures and higher demand for products. Similar findings were also reported by Chukwuma et al. (2021), Rinasti et al. (2022), and Tran-Thanh et al. (2022). Thus, road networks, facilities, and industries, which are the major producers of primary microplastics, were found to be responsible for microplastics leakage into waterways.

Among the three variable groups used in this study (i.e., primary, secondary, and background), primary sources of microplastics exert substantial influence on microplastics leakage due to their widespread distribution. These microplastics easily disperse from their production and usage points through various pathways, such as runoff, wind, and wastewater, ultimately reaching waterways. Conversely, secondary microplastics, originating from mechanical abrasion or degradation in landfill or dumpsites, enter river networks primarily through leachate. Consequently, primary microplastics have a higher likelihood of reaching river networks than secondary microplastics, highlighting the need to manage primary microplastic sources to mitigate microplastic pollution. Hence, implementing robust waste management strategies at primary microplastic production, distribution, and usage points is crucial for reducing environmental impact of microplastics.

Following the identification of the microplastics leakage dense areas, a microplastics leakage hotspots map was created to show the flow of microplastics from these areas to nearby river bodies. The hotspot map (Fig. 7) displays the potential flow of microplastics from the leakage-dense sub-districts of Ubon Ratchathani towards the primary urban river in the province, the Mun River. The map illustrates that the runoff flow direction, as indicated by the arrows, washes away the microplastics from the leakage-dense areas into the Mun River.

### Field verification of microplastics leakage source map

A field survey conducted in October 2019 reported presence of microplastics in the Mun River (four sampling: 0.08, 0.25, 0.68, and 0.49 pieces of microplastics per cubic meter), mostly comprising polypropylene (PP) and nylon, with traces of polyethylene (PE) and polystyrene (PS). This field-based study’s findings align with the hotspots map, underscoring the effectiveness of fuzzy logic-based GIS tools in identifying rivers susceptible to microplastics pollution within a region.

To further validate the reliability of the obtained hotspot map, the microplastics found in the Mun River were compared with those in the river outside the hotspot areas (Fig. 8). A microplastics pollution survey was conducted in the Phap River, located away from the leakage-dense areas and in proximity to three dumpsites and agricultural lands.

**Fig. 9:**
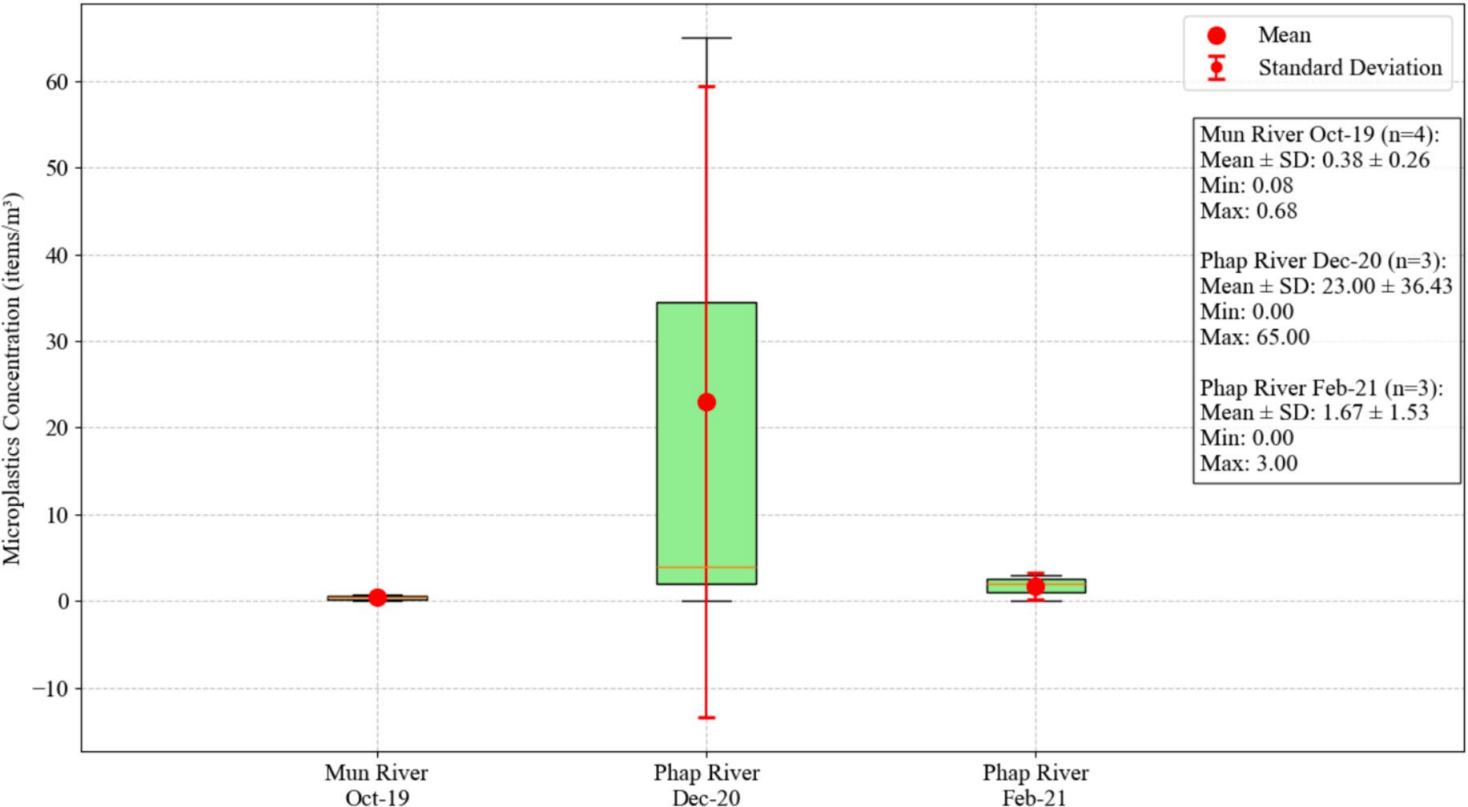
Comparative analysis of microplastics in Mun and Phap rivers

In December 2020, the three sampling sites in the Phap River had microplastic concentrations of 4, 0, and 65 microplastics per cubic meter, while only 0, 2, and 3 microplastics per cubic meter were found in the same sampling locations in February 2021. Both microplastics fragments and fibers were found in this river. The ATR-FTIR analysis identified specific bands, analyzed in accordance with Jung et al. (2018), such as CH bend and C–C stretch, aromatic CH out-of-plane bonds, and O–H bend, suggesting the presence of poly vinyl chlorides (PVCs), polystyrene (PS), and cellulose acetate, respectively, in the Phap River. The comparison details of the microplastics found in Mun and Phap river are shown in Fig. 9.

### Implications

#### Microplastics found in different rivers in Ubon Ratchathani

A comparison between the number of microplastics found in rivers inside and outside of leakage-dense areas, namely Mun and Phap rivers, respectively, showed a higher microplastic count in latter despite the former’s proximity to a higher number of microplastics sources, as shown in Fig. 9. The Mun River, situated in the urban center of Ubon Ratchathani and being the province’s major river, was expected to exhibit a higher susceptibility to microplastics, as indicated by the hotspot map. However, field-based sampling showed a higher number of microplastics in the Phap River, which is located in close proximity to agricultural lands and three dumpsites.

The sampling of microplastics in the Mun and Phap rivers was conducted during different months, corresponding to distinct seasons in Ubon Ratchathani. According to (WeatherSpark, 2022), in December, which marks the dry season in Ubon Ratchathani, the region typically experiences minimal runoff due to an average rainfall of only 1 mm. This could result in a higher accumulation of microplastics in the Phap River compared to February. This trend is supported by Rinasti et al. (2022), who reported increased plastic accumulation from the post-rainy season through the dry season, as observed in the Phap River, where the microplastic counts were high in December, followed by the wet season in August.

Moreover, the human activities near the Phap River are mostly agricultural, which is strongly influenced by seasonal variations. December corresponds to the rice harvesting season in Ubon Ratchathani (Food and Agriculture Organization of the United Nations, 2002), leading to high agricultural activities that can result in greater microplastics leakage into the Phap River. For the Mun River, sampling was conducted in October, a period when sporadic rainfall occurs in Ubon Ratchathani. This weather pattern allows transport of microplastics from the urban center of the province into the Mun River, as illustrated in Fig. 8. Additionally, the Phap River has a smaller flow rate than the Mun River, leading to a higher accumulation of microplastics.

#### Spatial dynamics and environmental impact of microplastics

The discrepancy between the Mun and Phap rivers highlights the influence of site-specific factors on the spatial distribution of microplastic leakage hotspots. These rivers exhibit distinct characteristics in terms of their surrounding land use patterns: the Mun River traverses an urbanized landscape characterized by extensive road networks and residential developments, while the Phap River flows through predominantly agricultural terrain with proximity to waste disposal sites. The temporal dynamics of human activities in these regions differ substantially. Urban activities adjacent to the Mun River maintain relative consistency throughout the year, whereas the Phap River’s surrounding agricultural activities demonstrate marked seasonal variations, with intensified human presence during cultivation periods. Furthermore, the rivers’ morphological and hydrodynamic characteristics vary significantly: the Mun River, being larger, exhibits higher flow rates, while the Phap River’s smaller dimensions result in reduced flow rates and distinct sediment transport capabilities.

Although urban environments typically generate higher concentrations of microplastic pollutants compared to agricultural areas, the distribution patterns (Fig. 9) suggest that river morphology and transport mechanisms, particularly those influenced by precipitation, play crucial roles in determining final microplastic concentrations. Thus, it can be deduced that the potential drivers of microplastics in Ubon Ratchathani that can be found in river networks are primary sources of microplastics (facilities such as restaurants, hotels, department stores/malls, road networks, construction, and transportation-based firms), land use patterns, seasonal weather variations, and river morphological characteristics including flow rates. This observation aligns with the findings of the previous study (Tran-Thanh et al., 2022) that developed a leakage pathway accumulation model for Ubon Ratchathani and Vientiane, highlighting the complexity of microplastics distribution in river systems.

The accumulation of microplastics significantly impacts water quality, thereby disrupting benthic habitats and aquatic organisms. The polar surfaces of microplastics can react with metal cations through non-specific interactions, facilitating the retention of heavy metals and increasing pollution on river surfaces (He et al., 2025; Holmes et al., 2012). These toxic substances can be absorbed or ingested by aquatic organisms, leading to adverse effects such as cell lysis, bioaccumulation, and oxidative stress (Dahms & Greenfield, 2025; Li et al., 2025). The Phap River, in particular, may face heightened ecological disturbances due to agricultural runoff during the critical rice planting season.

#### Policy recommendations

The identification of key drivers influencing microplastic distribution (primary sources, land use patterns, seasonal variations, and river morphology) provides a foundation for developing a comprehensive policy framework and multi-stakeholder engagement strategy. In urban areas, particularly those surrounding Mun River, the introduction of zonal regulations can help manage microplastics originating from areas with high-density of restaurants, shops, hotels, malls, and hospitals. Industrial regulations that promote eco-friendly practices and incorporate microplastic trapping at the source can significantly reduce leakage. Specifically for road networks, implementing roadside drainage collection systems and ensuring regular maintenance can help trap and reduce microplastic leakage.

For rural and agricultural regions, particularly in the vicinity of the Phap River, enhanced management strategies for agricultural runoff through the implementation of buffer zones and sustainable agricultural practices are essential. These measures should be complemented by strengthening waste management infrastructure across both rural and urban areas, with particular attention to preventing microplastic leakage from dumpsites. The technical aspect of monitoring and controlling microplastics leakage necessitates the deployment of remote sensing technologies and sensors for continuous real-time monitoring of microplastic distribution in water bodies, especially considering the influence of river morphology on pollution patterns. Furthermore, increasing public awareness and encouraging community-based initiatives focused on reducing plastic use and improving waste disposal behaviors are essential components of effective mitigation strategies. These policies may face significant barriers, including economic constraints, logistical challenges, and the need for infrastructure upgrades. Additionally, limited public awareness of microplastic pollution could hinder policy implementation. To address these challenges, a coordinated approach involving multiple stakeholders is essential. This necessitates collaborative efforts between government agencies responsible for policy formulation and enforcement, local communities ensuring implementation and compliance, industries driving technological innovation, and academic institutions conducting research and monitoring activities.

### Limitations of the study

Some limitations have been faced while conducting field-based study. Due to COVID-19 restrictions, a higher number of samplings could not be accomplished, which would have strengthened the verification of the hotspots map. The inability to conduct a broader sampling regime potentially limits the representativeness of the data, as fewer samples may not capture the full variability and distribution of microplastic leakage across different sites. This constraint could result in a less detailed verification process for the hotspots identified through remote sensing and GIS analyses. Consequently, while the existing data provides valuable insights, the limited field verification may affect the precision of the hotspot map and underscore the need for further validation in future studies.

Additionally, a hotspot map does not account the possible topographical constraints for sampling microplastics in the identified river networks, which also restricted the number of samplings of microplastics conducted in the rivers. These constraints pose significant challenges that limit the number and distribution of sampling locations, ultimately impacting the comprehensiveness of the data collected. Topographical challenges include steep terrain, dense vegetation, and inaccessible riverbanks, which can hinder the physical access required for effective sampling. Such obstacles not only make it difficult to reach certain sections of the river but also limit the deployment of equipment necessary for collecting samples across a wider area. The inability to conduct uniform sampling due to these geographical limitations may result in data gaps, potentially affecting the accuracy and representativeness of microplastics distribution assessments in these waterways. Consequently, while the hotspot map identifies key areas of concern, it does not fully capture the complex spatial variability influenced by these topographical barriers.

Furthermore, microplastics from various pathways, such as agricultural runoff, industrial runoff, and road networks runoff, were not sampled. A field study including sampling from these pathways will provide the trend of microplastics in these pathways, allowing identification of the sources that contribute highest to microplastics leakage. These pathways are significant conduits for microplastics entering aquatic ecosystems, and their exclusion represents a substantial gap in understanding the complete landscape of microplastic leakage. This can be useful in developing policies and mitigation strategies targeting specifically the highest contributing sources and their pathways.

Although hotspot maps identify river networks susceptible to microplastic pollution, they cannot provide quantitative assessment of types of microplastics present or pinpoint specific accumulation areas within the river networks. To achieve a comprehensive assessment, real-time and continuous monitoring of the microplastics in river networks situated within these leakage source hotspots should be conducted. The use of such maps to identify areas of concern regarding microplastic pollution should serve as an initial baseline, with the need for subsequent expansion through field-based collection.

### Recommendations

A comprehensive study of microplastics can be conducted in the areas highlighted by the hotspots map to understand the dynamics of microplastics pollution in the region. This would allow researchers to gain a deeper understanding of the dynamics of microplastic pollution in the region, including the spatial variability and concentration levels in these high-risk zones. Such studies could also reveal the interactions between microplastics and local environmental factors, such as hydrology and sediment types. Additionally, continuous monitoring of the river networks would enable the analysis of microplastics trends and its accumulation patterns across different seasons and sections of the river. Seasonal variations, influenced by factors such as rainfall and agricultural activities, can significantly affect microplastic levels. Furthermore, a detailed analysis of the pathways through which microplastics enter waterways, such as agricultural runoff, wastewater, and industrial discharges, can be done to identify the pathways that contribute most to microplastic leakage into the waterways. Understanding these pathways can inform targeted interventions, such as optimizing wastewater treatment processes or implementing sustainable agricultural practices, thereby mitigating their impact on waterway pollution.

## Conclusions

This study used a novel application of fuzzy logic-based GIS tools as an effective method for identifying potential sources of high microplastics leakage into waterways within a specific region. Focusing on Ubon Ratchathani, a province in Northeastern Thailand, this study successfully identified microplastics leakage hotspots by creating a microplastics leakage density map and then overlaying it with the drainage networks of the province. The leakage density map showed that the leakage of microplastics mostly focused on the urban sub-districts of the province due to presence of high primary producers of microplastics showing the need of microplastics pollution mitigation strategies to be focused on that part of the province. This study produced a comprehensive microplastics sources hotspot map, delineating the sub-districts of Ubon Ratchathani with high microplastic densities and illustrating their flow into Mun River. Substantiated through field-based microplastics survey, this study validates the fuzzy logic-based GIS tools as a reliable means of assessing microplastic pollution. It underscores the utility of these tools in pre-assessing the potential for microplastics leakage within a given region. Based on the obtained hotspots map, the local bodies can develop comprehensive strategies designed for the hotspot area which can include regular maintenance and implementation of stormwater management to capture microplastics from the runoff from the road networks. Additionally, enforcement of regulatory frameworks that include use of microplastics free raw materials and effective waste management in construction, transportation, electronics, and tires industries is also recommended. The study also suggests exploring more detailed microplastics sampling from industrial, agricultural runoff, and wastewater sources in future studies.

## Data Availability

Data is provided within the manuscript.

## Abbreviations

ATR-FTIR: Attenuated total reflection-Fourier transform infrared
DEM: Digital elevation model
ESRI: Environmental Systems Research Institute
GIS: Geographic information system
MCDM: Multi-criteria decision making
MSW: Municipal solid waste
PE: Polyethylene
PP: Polypropylene
PS: Polystyrene
PVC: Polyvinyl chloride
SDG: Sustainable Development Goals
